# DFT Study on the Substituent Effect of Anticancer Picoline-Diazido-Pt(IV) Compounds

**DOI:** 10.3389/fonc.2021.749178

**Published:** 2022-01-10

**Authors:** Meilin Mu, Hongwei Gao

**Affiliations:** School of Life Science, Ludong University, Yantai, China

**Keywords:** DFT, IR spectroscopy, geometry optimization, HOMO and LUMO, natural bond orbital, Pt(IV)

## Abstract

The geometric structure of azido Pt(IV) compounds containing picoline was calculated by using density functional theory(DFT) at the LSDA/SDD level. The ESP distribution shows the possible reaction sites of the compounds. In addition, the frequency calculation results assigned the infrared spectra of these compounds, and specified important stretching and bending vibrations. The HOMO-LUMO energy gaps of these compounds are also calculated to explain the charge transfer of the molecules. The distribution of Mulliken charges and natural atomic charges of these atoms is also calculated. Natural bond orbital(NBO) analysis explains the intramolecular interactions and their electron density.

## 1 Introduction

Metal platinum therapy is usually a cancer treatment plan including cisplatin (1), carboplatin (2, 3) and oxaliplatin (4). Especially the successful medical treatment of cisplatin can be called a milestone in cancer chemotherapy. But the incidental properties of these three platinum drugs include poor drug resistance, side effects and targeting (5, 6). In order to solve the problems of these drugs, new drug designs have been implemented to obtain better therapeutic effects. One of the solutions involves introducing new groups into the system to become Pt(IV) to give the compound new stability and new functional groups (7, 8). Drug improvement based on cisplatin (9, 10) and carboplatin (11) are also promising directions to improve the properties of the parent compounds. In addition, a class of compounds can obtain active reactants through light-induced activation, which may be a new direction for targeted therapy. Photodynamic therapy(PDT) is a kind of light therapy that achieves therapeutic effects by producing highly reactive singlet oxygen ^1^O_2_ (12). However, the tumor affected by hypoxia is a barrier that is not easy to recover for oxygen-dependent PDT (13). Photoactivated chemotherapy (PACT), a light therapy that is not limited to oxygen, is considered a promising therapy. Some members of transition metals often become prodrugs of PACT (14, 15). The compounds suitable for this method are not limited by the oxygen concentration, and can form a reactive active substance after being irradiated (16).

As a new type of anti-tumor platinum drug, Pt(IV) compounds containing azido have excellent stability in the dark, and they involve the release of azido groups and the production of reactive Pt(II) reactants after being irradiated (17, 18). In addition after these compounds have been developed, they can obviously overcome the easy-to-reducible properties of diiodide Pt(IV) compounds (19). The introduction of new groups has become an effective way to improve the performance of azido-containing compounds. One of the transformation methods for cisplatin, namely the introduction of heterocyclic imines(especially pyridine), has been found to have significant damaging capabilities for cancer tissue (20, 21). In addition, the way *cis*-diammine-(pyridine)-chloroplatinum(II) (or cDPCP) binds to DNA and the way it inhibits transcription are different from drugs such as cisplatin (22). Such monofunctional compound provides an excellent idea for appropriately improving the pharmacological activity of drug such as cisplatin.

In previous studies, we have introduced and classified the mechanism of action and toxicity of azido-containing Pt(IV) complexes in detail (23). The addition of the aromatic group(py) can significantly increase the phototoxicity of the compound *trans,trans,trans*-[Pt(N_3_)_2_(OH)_2_(NH_3_)(py)] compared with the amine-based compound *trans,trans,trans*-[Pt(N_3_)_2_(OH)_2_(NH_3_)_2_] (24, 25). The single pyridine compound *trans,trans,trans*-[Pt(N_3_)_2_(OH)_2_(NH_3_)(py)] is non-selective to a variety of cisplatin cells. Moreover, the photoactivation process of *trans,trans,trans*-[Pt(N_3_)_2_(OH)_2_(NH_3_)(py)] and DFT/TDDFT calculations prove that its reaction involves the drop of azide ligand (26). *Trans,trans,trans*-[Pt(N_3_)_2_(OH)_2_(py)_2_](1) is a promising candidate, which can bind to DNA firmly after being activated, and can show much higher photocytotoxicity than cisplatin (27). Furthermore, compound 1 tends to have higher intracellular accumulation and photocytotoxicity compared with its monopyridine azido compound (28). The picoline analog *trans*,*trans*,*trans*-[Pt(N_3_)_2_(OH)_2_(R_1_)(R_2_)] of compound 1 also have excellent phototoxicity to cisplatin-resistant cells and other specific cell lines. They are the compounds 2(R_1_ = 2-picoline, R_2_=pyridine), 3(R_1_ = 3-picoline, R_2_=pyridine), 4(R_1 =_ 4-picoline, R_2_=pyridine), 5(R_1_ = 3-picoline, R_2_ = 3-picoline) and 6(R_1_ = 4-picoline, R_2_ = 4-picoline) (29).

Generally speaking, we hope that Pt(IV) diazonium compounds are stable in the dark and have cytotoxic effects after being reduced to Pt(II) compounds. For Pt(IV) compounds, their stability is a very important criterion for evaluating whether a compound is excellent when it is not reduced to Pt(II). Therefore, our manuscript focuses more on the stability of tetravalent complexes. Although there have been many experimental studies on such compounds, there are few theoretical studies on these compounds (30). Previous studies have shown that long-range corrected functional, LC-ωPBE, exhibits good characteristics in DFT calculation of cisplatin geometry optimization and Pt ligand vibration. In addition, PBE0 and mPW1PW functional combined with the LanL2TZ(f) basis set of Pt can be used to calculate the IR and Raman spectra of cisplatin (31). Compared with the experimental data, the B3LYP/(aug-cc-pVDZ + GD3BJ) method can predict the vibrational spectra of carboplatin, oxaliplatin, nedaplatin and heptaplatin well (32). We have optimized the geometric structure of compound 1 and analyzed the vibration spectrum before (33). In addition, the HOMO-LUMO orbital, natural bond orbital (NBO) and charge distribution of the compounds are also analyzed.

## 2 Computational Details

In density functional theory, Local Spin Density Approximation (LSDA) will underestimate the band gap value, and it is not suitable for systems with rapidly changing electron densities. The Generalized Gradient Approximation (GGA) often includes the gradient correction as the LSDA correction (34, 35). Hybrid functionals can better describe systems with more serious electronic self-interactions. Hybrid functionals can adjust the ratio of HF exchange functionals and DFT exchange functionals by changing the linear combination coefficients, so that the calculated results are closer to the real results of the experiment.

In previous research, We screened some functional methods and basis sets in Gaussian 16 that are suitable for pyridine-containing azido Pt(IV) complexes (33). When calculating the geometric optimization data, the functionals with smaller deviations from the experimental data are CAM-B3LYP, PBE1PBE, LSDA, etc. But we found that the LSDA functional method has small errors with the experimental values on the key bond length data of Pt-N(N_3_), Pt-O(OH) and N(N_3_)-N(N_3_). In the same way, although LANL2DZ (36) also has a small deviation, the calculation results of the SDD basis set show that bond lengths [Pt-N(N_3_), Pt-O(OH) and N(N_3_)-N(N_3_)] fit well. Therefore, we choose LSDA functional and SDD basis set as our calculation method. All calculation results are obtained at the local spin-density approximation(LSDA) (37, 38) & Stuttgart/Dresden and D95 ECPs(SDD) (39, 40) level of the density functional theory (DFT) method in Gaussian 16 (41) software. Firstly, the molecular structure of the compounds was optimized to analyze the structural characteristics of the compounds. The optimized structure is used for frequency calculation, and their characteristic absorption peaks are assigned. Electrostatic potential (ESP) is considered to be a possible method to predict the location of the interaction reaction. The ESP distribution of the compounds is drawn by Multiwfn program (42) and Visual Molecular Dynamics 1.9.3 (VMD 1.9.3) program (43). In addition, electronic properties such as HOMO-LUMO energy, Mulliken charge distribution and natural bond orbital (NBO) and natural atomic charge are calculated and analyzed.

## 3 Results and Discussion

### 3.1 Cytotoxic Activity

For the structure of the selected compounds 1, 2, 3, 4, 5 and 6, the main difference lies in the different positions and different amounts of picoline. In this article, we will discuss the effect of substituents on the properties of the compounds. Previous studies have completed the toxicity testing of these compounds through experiments, and their toxicity data is shown in **Table 1** (29). Except for compound 2 which exhibits moderate toxicity in the dark, other compounds have good stability in the dark. In addition, compound 2 also has poor phototoxicity (14.5μM in human ovarian cells), and other compounds have good phototoxicity IC_50_ values (3.1-7.2 μM in human ovarian cells). In addition, compared with compound **5** (10.4 μM), compounds 3 and 4 showed better phototoxicity(3.3 and 4.6 μM) for the cisplatin-resistant cell line (A2780cis). Compared with its analogs 4 and **5**, compound **6** has good cytotoxicity among the selected compounds (44).

**Table 1:** Cytotoxicity of compounds 1, 2, 3, 4, 5 and 6 in A2780 and cisplatin-resistant cells(A2780cis) (29).

compound	IC_50_ [μM] (420 nm)	
	A2780	A2780cis
**1**	6.7	^a^ DN
**2**	14.5	15.5
**3**	4.0	3.3
**4**	5.4	4.6
**5**	7.2	10.4
**6**	2.1	4.1

a DN, not determined.

### 3.2 Molecular Geometry and ESP Analysis

**Figure 1:**
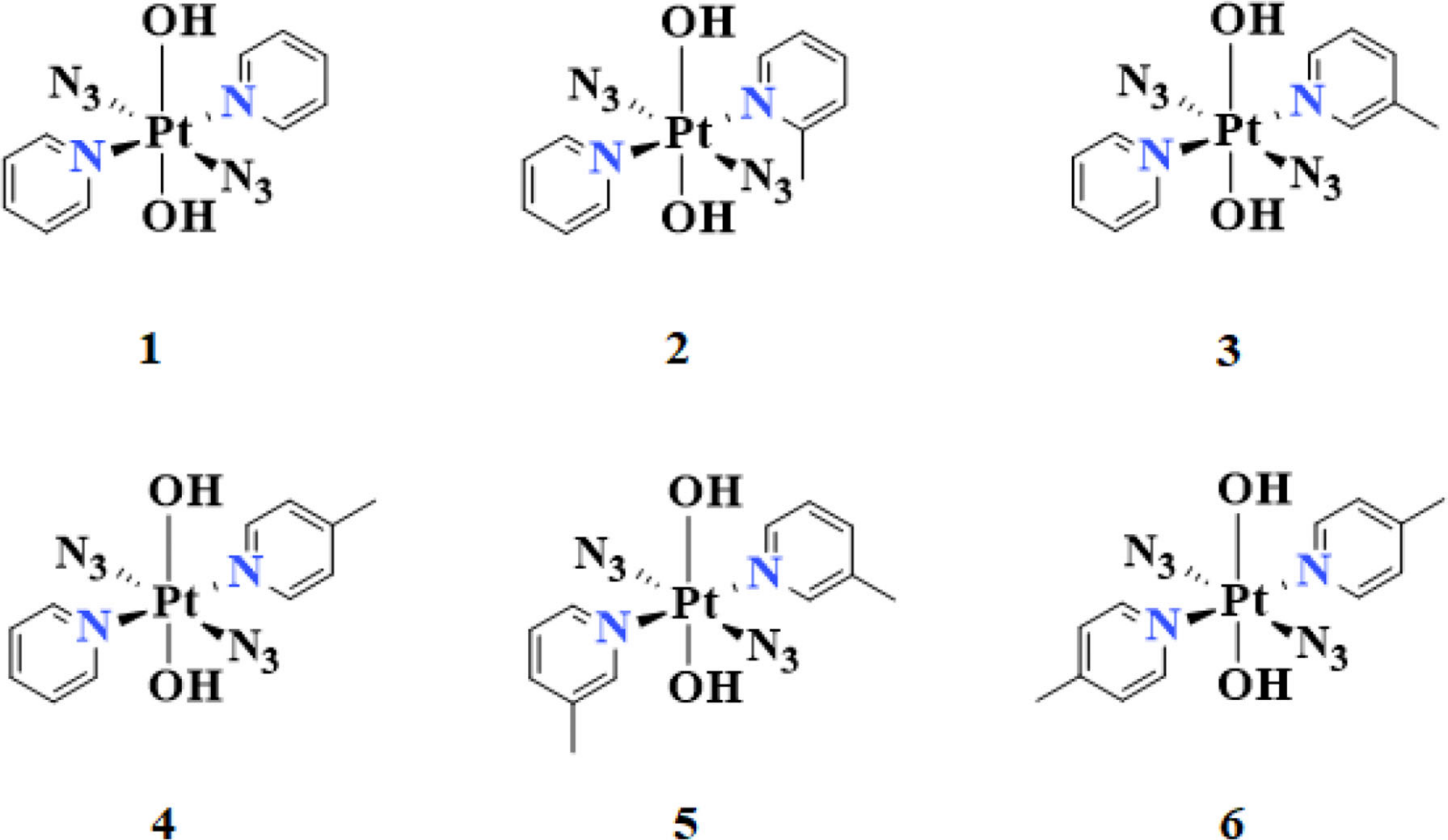
The calculated chemical structures of compounds: *trans*,*trans*,*trans*-[Pt(N_3_)_2_(OH)_2_(R_1_)(R_2_)][2(R_1_=2-picoline, R_2_=pyridine), 3(R_1_=3-picoline, R_2_=pyridine), 4(R_1_=4-picoline, R_2_=pyridine), 5(R_1_=3-picoline, R_2_=3-picoline) and 6(R_1_=4-picoline, R_2_=4-picoline)].

**Figure 2:**
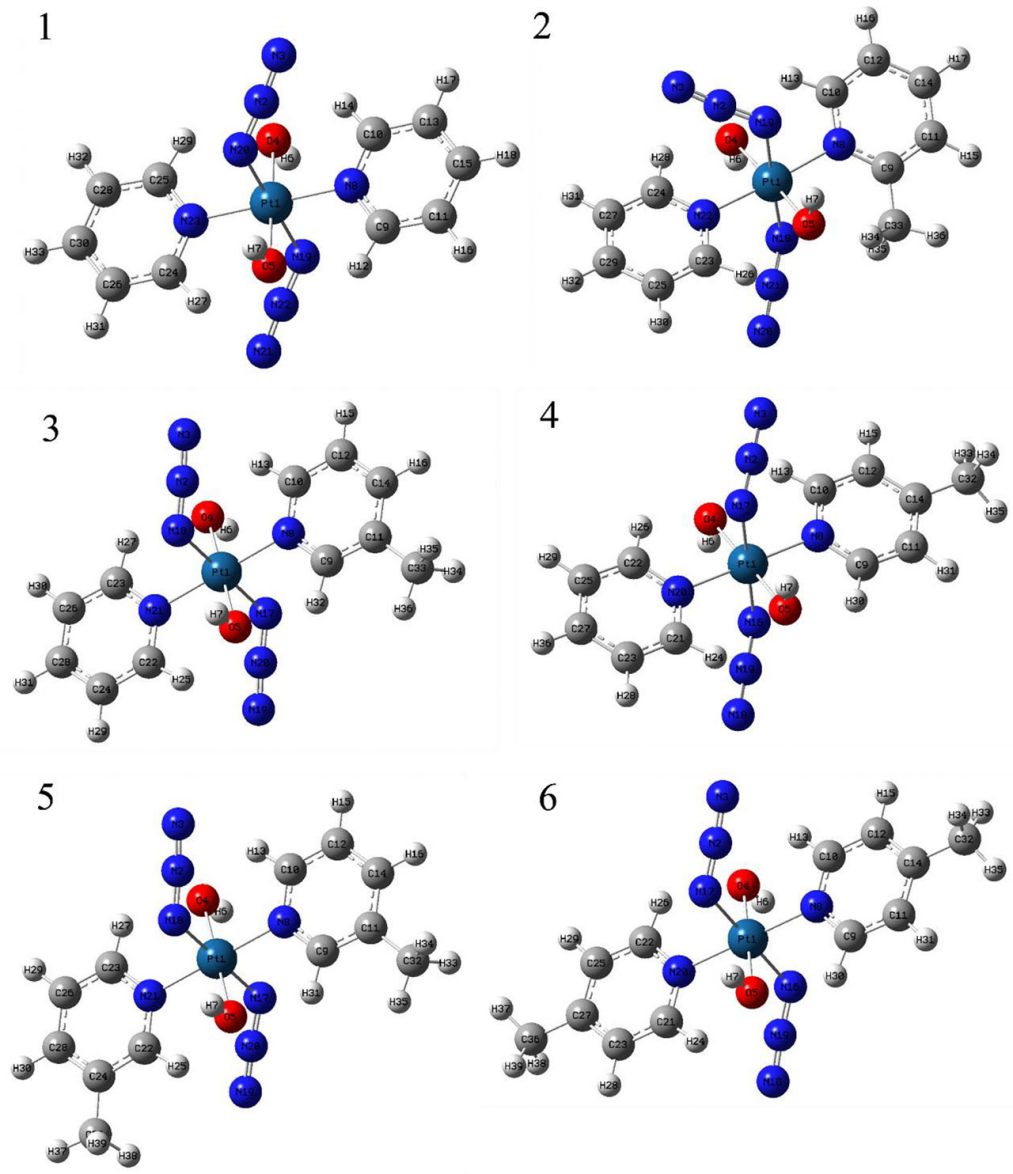
The optimized structure of compounds 1, 2, 3, 4, 5 and 6.

The chemical structures and optimized molecular structures of the selected compounds 1, 2, 3, 4, 5 and 6 are shown in **Figures 1** and **2**. For the compounds, the leaving group N_3_ is usually the primary research object due to its importance in the first reaction stage, and the release rate of the N_3_ group is the reference feature for the rate of photoactivation (18, 45). The OH group is the key group when the compounds and nucleosides are combined, and the non-leaving group containing pyridine will significantly affect the photocytotoxicity of the compounds (24, 46). We have optimized their structure on the LSDA/SDD level, and the optimized structure data of the bond length and bond angle are summarized in **Table 2**. The date is used to evaluate the possible influence of the substituent effect on the stability and cytotoxicity of the compounds. We compared the calculated dates of complexes 1, 3, 4, 5 and 6 with the experimental values. The results show that their calculated bond length data is relatively close to the experimental data. In this part only, we have uniformly numbered the structures of the compounds for the convenience of research and discussion (**Figure S1**).

**Table 2:** Optimized bond length(in Å) and bond angle(in deg) of compounds 1, 2, 3, 4, 5 and 6.

Geometry	Exp.	1	2	3	4	5	6
R(Pt1-O1)	2.027	2.017	2.013	2.016	2.017	2.017	2.018
R(Pt1-O2)	1.990	2.017	2.027	2.017	2.017	2.019	2.018
R(Pt1-N1)	2.046	2.033	2.036	2.033	2.033	2.037	2.033
R(Pt1-N4)	2.043	2.033	2.036	2.032	2.033	2.030	2.033
R(Pt1-N7)	2.047	2.004	2.025	2.005	2.004	2.006	2.004
R(Pt1-N8)	2.046	2.004	2.016	2.005	2.005	2.006	2.004
R(N5-N6)	1.146	1.187	1.187	1.187	1.187	1.187	1.187
R(N4-N5)	1.128	1.243	1.244	1.243	1.243	1.243	1.243
R(N1-N2)	1.215	1.243	1.243	1.243	1.243	1.242	1.243
R(N2-N3)	1.140	1.187	1.188	1.187	1.187	1.188	1.187
R(N8-C3)		1.353	1.354	1.353	1.353	1.350	1.353
A(N1-Pt1-N4)		180.0	176.7	179.9	179.9	179.4	180.0
D(Pt1-N1-N2-N3)	175.7	179.8	-178.2	179.6	179.6	177.5	179.5
D(Pt1-N4-N5-N6)	177.9	-179.7	-178.4	-179.1	-179.5	-180.0	-179.5
D(N2-N1-N4-N5)	176.8	-180.0	-77.6	-179.9	177. 6	-170.9	180.0
D(C1-N8-N7-C2)	119.5	-1.4	22.1	-0.9	-2.1	0.2	-1.5

The poorer cytotoxicity of compounds 2 and 4 than other compounds may be related to the bond length of the Pt-N bond. Previous studies have shown that after the introduction of 2-picoline, compound 2 has the fastest reduction rate among our selected compounds (29). The steric clash between the methyl group of 2-picoline and the axial hydroxyl ligand makes the compound 2 is easier to be restored (47). The data in **Table 2** shows that the Pt-N_3_ bond lengths (Pt1-N1 and Pt1-N4) of compound 2 are 2.036 and 2.036 Å, respectively. The Pt-N_3_ bond lengths of compounds 1, 3, 4, 5 and 6 are 2.032-2.033 Å. Therefore, the results show that the binding ability between the Pt-N bonds between the Pt and the azido group in the compound 2 is weaker than that of the compounds 3, 4, and 6, which indicates that the azido group is easily released from the compound 2 after being irradiated by light. Because the photoreaction of the azido compound involves the reduction of Pt(IV) to Pt(II) and the drop of N_3_ after being irradiated. In addition, the Pt1-N1 and Pt1-N4 bond lengths of the compound **5** are 2.037 and 2.030 Å, which are the longest and shortest bond lengths respectively compared with other compounds. That is to say, the binding ability of the Pt atom and the N1 atom of the compound **5** is very weak, and the binding ability to the N4 atom is very strong compared with other compounds. Therefore, according to the previous report, the two azido groups of the compound 5 may be released one by one to participate in the photolysis pathway, and the second N_3_ group may be released after binding to the nucleotide (46).

It has been confirmed that the production of O_2_ in the photoproduct is likely to come from the hydroxyl groups of such compounds, and the decomposition of these hydroxyl groups after recombination is the key to the release of oxygen (46, 48). The results of the bond length between Pt atom and O atom show that the bond lengths of Pt-O1 and Pt-O2 of compound 2 are 2.013 and 2.027 Å, while the Pt-O bond lengths of the other compounds are relatively equal (2.017-2.019 Å). Therefore, the binding ability of the Pt-O2 bond of the compound 2 is weaker than other compounds, and the binding ability of the Pt-O1 of the compound 2 is less than other compounds.

An important event for the inactivation of cisplatin in cells is the replacement of amines, which affects the activity of the drug directly (49). The azido Pt(IV) compounds, *trans*,*trans*,*trans*-[Pt(N_3_)_2_(OH)_2_(NH_3_)_2_] & *trans*,*trans*,*trans*-[Pt(N_3_)_2_(OH)_2_(NH_3_)(py)], both have the dissociation of NH_3_ after being illuminated (24, 50). The pyridine group of the new compound 1 has not been observed to be released and has high cytotoxicity, so the addition of the pyridyl group can compensate for the loss of phototoxicity after the amino group is replaced (51). The bond lengths of Pt1-N7 and Pt1-N8 of the selected compound 2 are 2.025 and 2.016 Å, respectively, which are longer than the bond lengths of Pt-N(pyridine) bonds in other compounds (2.004-2.006 Å). In other words, the binding capacity of the compound 2 with Pt atoms is much smaller than that of other compounds. Therefore, according to previous studies, the stability of the Pt-N bond between the Pt atom and the pyridine group is directly related to the phototoxicity of the compounds. As shown in the data in **Table 1**, compound 2 has poor phototoxicity compared with other compounds, which is consistent with our calculation results.

We have compared compounds 2, 3 and 4 with different substitution positions, and the results show that meta-picoline compound 3 and para-picoline compound 4 are basically the same. For Ortho-picoline compound 2, although the bond length of Pt-O1 is smaller than that of compounds 3 and 4, the bond lengths of Pt-O2, Pt1-N1, Pt1-N4, Pt1-N7, and Pt1-N8 are all longer than compounds 3 and 4. Therefore, for single pyridine compounds with different substitution positions, the substituent effect is more significant on compound 2. And the bond length of compound 2 is mostly longer, which may be related to its poor phototoxicity and dark stability. For the effect of different numbers of picoline substituents, we have analyzed the bond lengths of compounds 1, 4 and 6, and the results show that their bond length data are not much different. The bond length analysis results of compounds 1, 3 and 5 show that the bond length data of compounds 1 and 3 are basically the same. The bond lengths of Pt1-N4 and N8-C3 of compound 5 are slightly smaller than that of compounds 1 and 3, and the bond lengths of Pt-O2, Pt1-N1, Pt1-N7 and Pt1-N8 are all longer. Moreover, the cytotoxicity of compound 5 is relatively small, so the number of substituents may also have a significant impact on cytotoxicity.

In addition, the two pyridine/picoline ligands of compound 2 have a large angular deflection (dihedral angle of 22.1°), which may be caused by the steric hindrance of compound **2**. Among the six types of compounds, the bond lengths of Pt-N(N_3_), Pt-OH and Pt-N(pyridine) mentioned above are in the order of Pt-N(N_3_)>Pt-OH>Pt-N(pyridine). Therefore, the order of the binding ability between Pt and adjacent groups is the pyridine(non-leaving group)>OH>N_3_(leaving group) for the compounds we selected. The above analysis is consistent with the results shown by the mechanism of action of these compounds.

**Figure 3:**
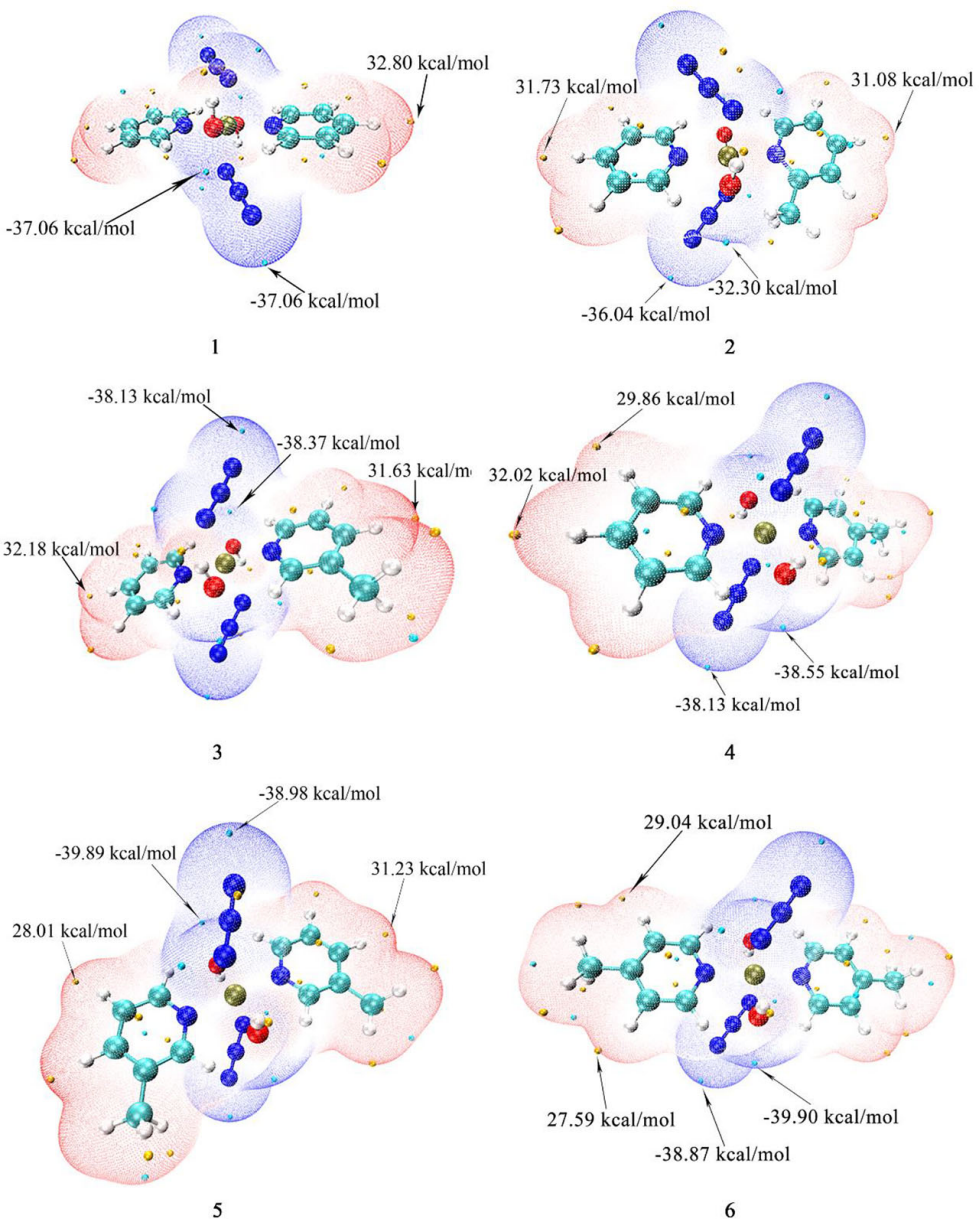
The calculated electrostatic potential of compounds 1, 2, 3, 4, 5 and 6.

The surface electrostatic potential (ESP) can provide a reference for the interaction mode between molecules, and the interaction between molecules depends on the ESP extreme point of the binding site (52–57). The extreme values of the positive and negative potentials drawn with Multiwfn and VMD are shown in **Figure 3**. In the figure, the ESP value of the blue area is negative, and the red area is positive. The para-position of the N atom in pyridine/picoline in all compounds is the most vulnerable site to nucleophilic attack, and the maximum ESP value comes from compound 1 (32.80 kcal/mol). Compound 6 has a methyl substituent at the para position, which causes its extreme point to shift to the meta H position. The N_3_ and OH^-^ regions of all compounds have one or more ESP minimums, which are the most vulnerable to proton attack. The lower ESP values are -39.89 and 39.90 kcal/mol for compounds 5 and 6.

For the mono-substituted compounds 2, 3 and 4 with different substitution positions, the minimum points of ESP are -36.04, -38.37 and -38.55 kcal/mol, respectively. So the para-compound 4 has the smallest extreme point for different substituent positions. For 3-picoline compounds 1, 3 and 5, their minimum ESP values are -37.06, -38.37, and -39.89 kcal/mol, respectively. For 4-picoline compounds 1, 4 and 6, their minimum ESP values are -37.06, -38.55 and -39.90 kcal/mol, respectively. Therefore, the ESP value results of the compounds with different numbers of substituents show that the minimum value of ESP decreases due to the increase of the number of substituents.

### 3.1 Vibrational Assignments

**Figure 4:**
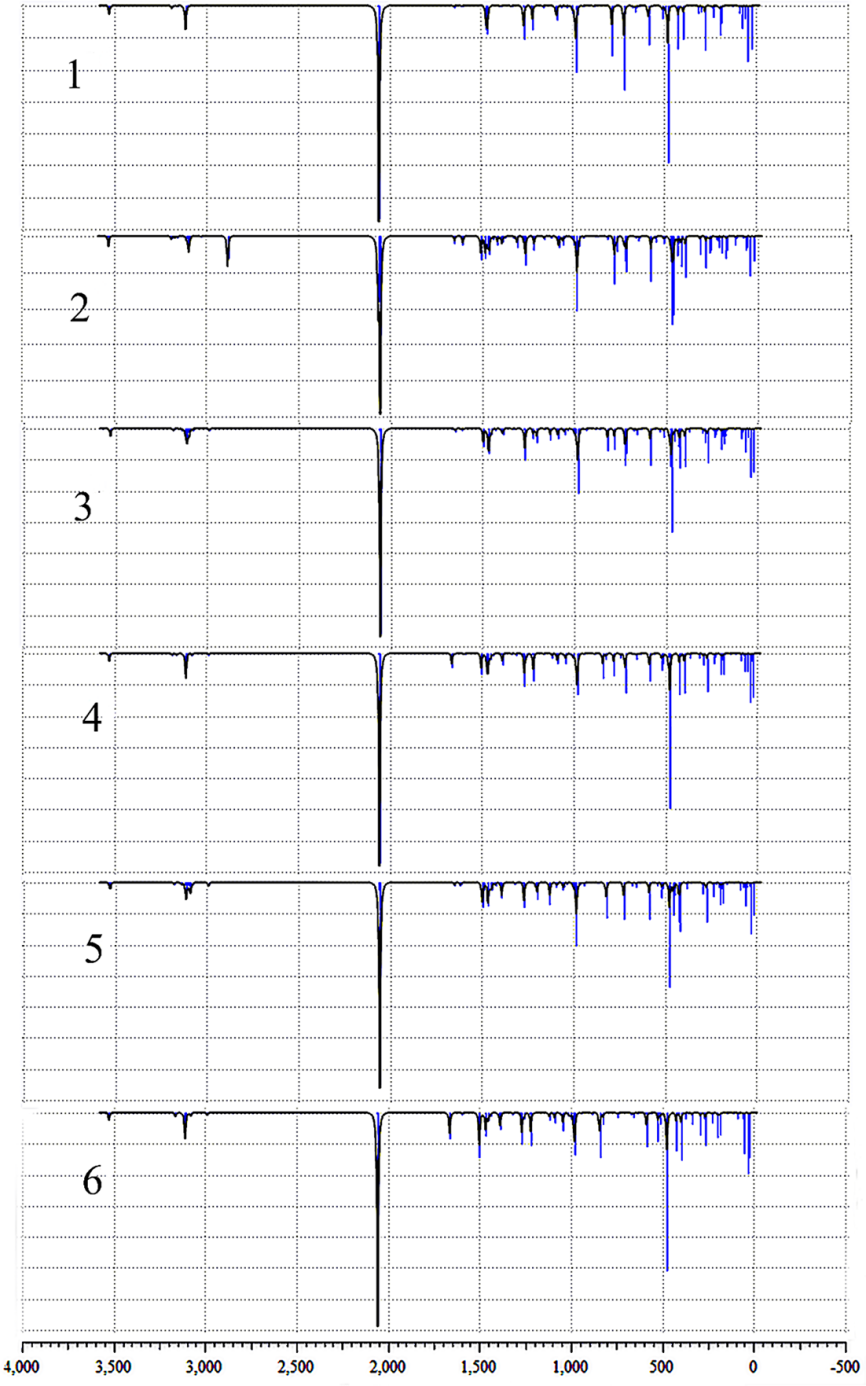
The calculated infrared spectra of the compounds 1, 2, 3, 4, 5 and 6.

The optimized structure of the selected compounds has been shown in **Figure 2**. The infrared characteristic peaks of different compounds have been assigned, and **Figure 4** has shown their infrared vibration spectra. The infrared vibration frequencies and corresponding vibration intensities of some important groups are summarized in **Table 3**. All vibrations are obtained by optimized calculations at the LSDA/SDD level. It is worth mentioning that in addition to the strong azido group’s stretching vibration intensity, the vibration modes of other groups also have high peak intensity. Among the selected vibration modes, stretching vibration and bending vibration are the most active vibration modes.

**Table 3:** Frequencies(cm-1) and infrared intensitiesa(km/mol) calculated for compounds 1, 2, 3, 4, 5 and 6.

Assignment^b^	1	2	3	4	5	6
υ_asym_ (N_3_–Pt–N_3_)	424 (40.1)	420 (23.6)	425 (38.5)	424 (39.3)	425 (38.3)	425 (37.2)
δ (OH)	480 (168.9)	468 (80.2)	471 (112.0)	477 (168.9)	475 (113.3)	475 (172.3)
υ_sym_ (HO-Pt-OH)	566 (0.0)	558 (5.5)	564 (0.6)	565 (0.0)	563 (0.5)	564 (0.0)
υ_asym_ (HO-Pt-OH)	587 (50.5)	584 (50.6)	587 (49.6)	587 (35.6)	587 (48.6)	588 (45.5)
γ (py)	785 (86.5)	782 (71.1)	781 (35.9)	781 (37.3)	820 (64.8)	843 (86.2)
υ_sym_ (N_3_)	1267 (92.5)	1265 (69.5)	1268 (89.4)	1267 (91.8)	1268 (69.2)	1268 (91.1)
δ (py)	1471 (76.0)	1470 (35.3)	1469 (35.8)	1470 (62.0)	1497 (83.3)	1466 (76.4)
δ (py)	1646 (3.9)	1649 (19.9)	1648 (8.3)	1662 (50.5)	1648 (9.0)	1662 (97.6)
υ_asym_ (N_3_)	2056 (982.1)	2052 (684.6)	2057 (969.4)	2056 (987.7)	2056 (952.2)	2056 (998.3)
υ_sym_ (CH_3_)	—	2880 (123.1)	2990 (10.7)	2987 (6.1)	2990 (11.1)	2987 (3.6)
υ_asym_ (CH_3_)	—	3026 (3.0)	3078 (5.7)	3077 (8.6)	3078 (6.1)	3077 (12.5)
υ_sym_ (CH_py_)	3111 (97.5)	3092 (61.7)	3110 (53.9)	3110 (92.0)	3113 (65.6)	3109 (96.1)
υ_asym_ (OH)	3527 (39.5)	3527 (41.0)	3527 (36.5)	3526 (37.7)	3526 (28.0)	3525 (36.8)

^a^IR intensity: the value in parentheses. ^b^Vibrational modes: *v* = stretch; scr = scissoring; γ = out-of-plane angle bending, δ = in-plane deformation, sym = symmetric and asym = asymmetric. Out-of-phase and in-phase notations refer to the phase between dissimilar vibrations or analogous vibrations when more than one of the same moieties exists.

#### 3.3.1 Hydroxido Vibrations

In all the compounds, the asymmetric stretching vibration υ_asym_(OH) of the hydroxyl group was observed at 3525-3527 cm^-1^, which is basically consistent with the previously measured OH stretching vibration (58, 59). In addition, OH in-plane deformation vibration δ(OH) was observed at 468-480 cm^-1^. After the pyridine group of compound 1 was replaced by picoline, the positions of the other complexes only shifted slightly (compounds 2 and 3 shifted around 10 cm^-1^, the compounds 4, 5 and 6 shifted around 5 cm^-1^). For the asymmetric stretching vibration υ_asym_(HO-Pt-OH) between the metal Pt atom and the hydroxyl ligand, compound 2 was observed to appear at 584 cm^-1^, and the compounds 1, 3, 4, 5, 6 were observed at 587-588 cm^-1^. Therefore, in this vibration mode, the differently substituted picoline has little effect on the infrared absorption position. The symmetric stretching vibration υ_sym_(HO-Pt-OH) between the metal Pt atom and the hydroxyl ligand is observed at 263-566 cm^-1^ (except for compound 2 at 558 cm^-1^). However, it is worth noting that the infrared absorption intensity of compounds 1, 2, 3, 4, 5, 6 in the υ_sym_(HO-Pt-OH) vibration mode is not strong.

#### 3.3.1 Azido Vibrations

The azido asymmetric stretching vibration υ_asym_(N_3_) has the strongest absorption intensity peak in all vibration modes. Except for the slight shift of the wavenumber of compound 2 (2052 cm^-1^), the calculated wavenumbers of other compounds are all around 2056 cm^-1^. Another vibration mode of the azido group is symmetric stretching υ_sym_(N_3_), and their absorption peaks are observed at 1265-1268 cm^-1^. In addition, the υ_asym_(N_3_–Pt–N_3_) absorption peaks of compounds 1, 3, 4, 5, 6 were observed at 420-425 cm^-1^.

The azido asymmetric stretching vibration υ_asym_(N_3_) has the strongest absorption intensity peak in all vibration modes. Except for the slight shift of the wavenumber of compound 2 (2052 cm^-1^), the calculated wavenumbers of other compounds are all around 2056 cm^-1^. Another vibration mode of the azido group is symmetric stretching υ_sym_(N_3_), and their absorption peaks are observed at 1265-1268 cm^-1^. In addition, the υ_asym_(N_3_–Pt–N_3_) absorption peaks of compounds 1, 3, 4, 5, 6 were observed at 420-425 cm^-1^.

#### 3.3.3 Pyridine Vibrations

There have been previous studies on the vibrational spectroscopy of pyridine compounds bound to different metals (60, 61). There are many types of in-plane deformation vibration of pyridine groups δ(py), one of which is that the wavenumbers of compounds 1, 2, 3 and 5 are 1646-1649 cm^-1^. However, the corresponding vibration peaks of the two 4-picoline compounds 4 and 6 have a similar shift, and their peaks are observed at 1662 cm^-1^ and 1662 cm^-1^. Therefore, for compounds 1, 4 and 6 with different amounts of 4-picoline, the absorption wavenumber of these compounds increased after adding 4-picoline. For another δ(py) vibration, the wavenumbers of the other compounds are concentrated at 1466-1471 cm^-1^ except for the deviation of compound 5(1497 cm^-1^). The reason for the shift is that the methyl of the compound 5 is working, and the similar wavenumber is observed in the δ(3-pic) of the compound 3. For the out-of-plane angle bending vibration γ(py) of the pyridine group, the vibrations of compounds 1, 2, 3, 4, 5 are observed to be distributed at 781-785 cm^-1^, and the vibrations wavenumber of compounds 5 and 6 are respectively observed at 820 cm^-1^ and 843 cm^-1^. Therefore, for the influence of picoline substitution on the wavenumber of γ(py), the unsubstituted and monosubstituted compounds only have a small deviation, but the disubstituted compounds 5 and 6 have a larger deviation. For the wavenumber of γ(py) vibration, unsubstituted compound=mono-substituted compounds<di-substituted compounds, the wavenumber of double-substituted compounds has increased a lot. That is because the methyl group is added to the unsubstituted pyridine, which increases the wavenumber of the out-of-plane bending vibration of the compounds 5 and 6.

#### 3.3.4 Methyl Vibrations and C-H Vibrations

For the compounds 2, 3, 4, 5 and 6, the introduction of picoline allowed the methyl characteristic absorption υ_sym_(CH_3_) and υ_asym_(CH_3_) to be observed. The wavenumber of the υ_sym_(CH_3_) of compound 2 was observed at 2880 cm^-1^, and the wavenumbers of the other compounds (2987-2990 cm^-1^) shifted by about 100 cm^-1^. In addition, the infrared absorption intensity of the compound 2 is higher than other compounds for this vibration mode. For the methyl asymmetric stretching vibration υ_asym_(CH_3_) on the pyridine group, the wavenumbers of compounds 2, 3, 4, 5, and 6 are observed at 3026, 3078,3077, 3078, and 3077 cm^-1^, respectively. The two C-H bonds connected to the N atom on the pyridine have a strong infrared absorption intensity in their symmetric stretching vibration υ_sym_(CH_py_). The absorption wavenumber of compound 2 is 3092 cm^-1^, while the compounds 1, 3, 4, 5, 6 have no significant shift (3109-3113 cm^-1^). The υ(CH_3_) vibration wavenumbers of compounds 2, 3, 4, 5, and 6 have been increased after the CH_3_ group forms a bond with pyridine. However, due to the steric hindrance of the compound 2, the vibration wavenumber of υ(CH_3_) of the compound 2 is smaller than that of compounds 1, 3, 4, 5, 6. Therefore, the vibrational spectral distribution of the ortho-picoline compound 2 is most affected by the substituent effect.

### 3.4 HOMO–LUMO Analysis

**Figure 5:**
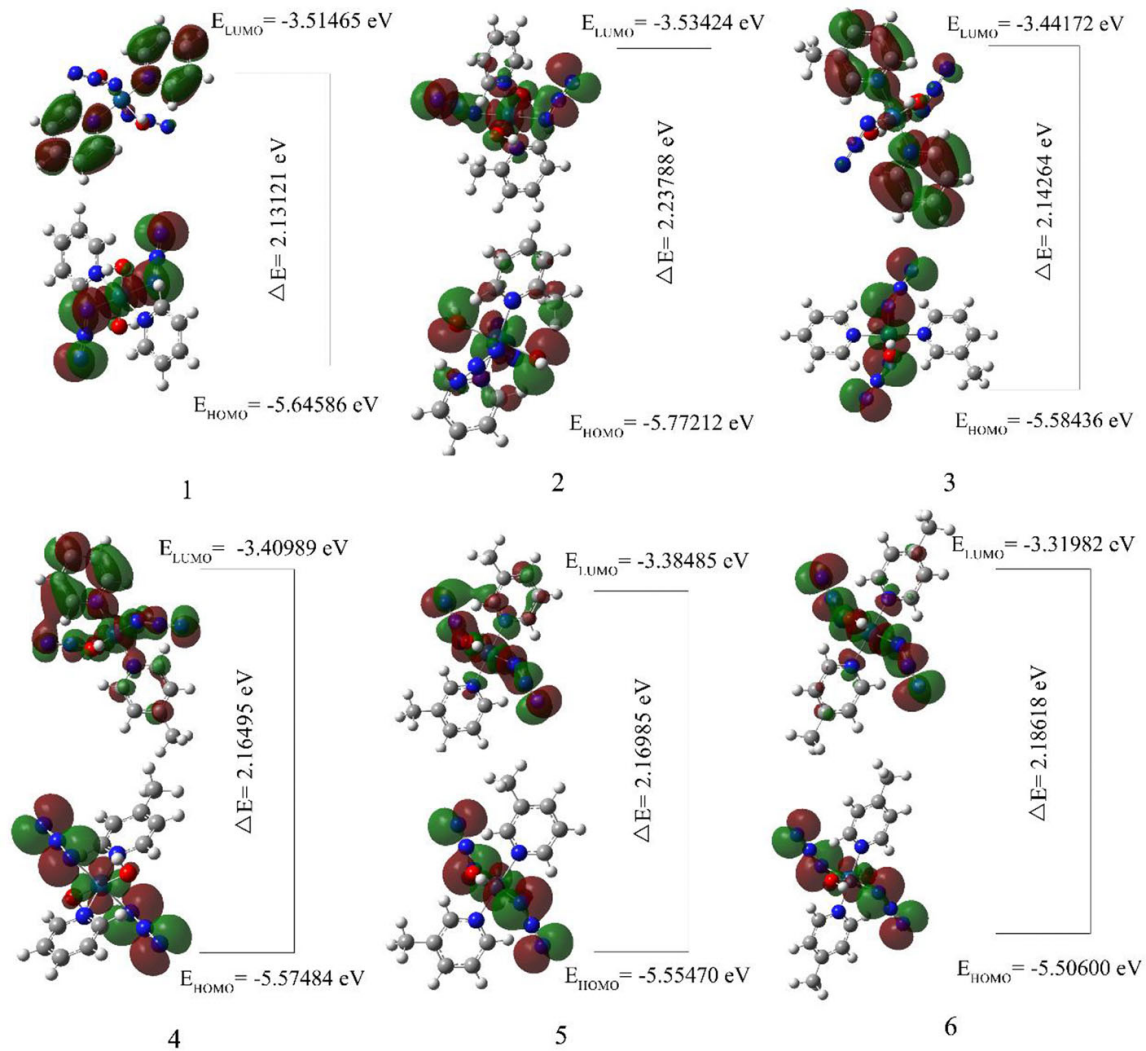
The calculated molecular orbital HOMO-LUMO of compounds 1, 2, 3, 4, 5 and 6.

The highest occupied molecular orbital (HOMO) and the lowest unoccupied molecular orbital (LUMO) respectively represent the ability to provide electrons and the ability to obtain electrons (62). The energy gap between HOMO and LUMO determines the charge transfer effect in the molecule (63, 64). The energy gap of the compounds selected in this article is calculated using the LSDA/SDD level (**Table 4**, **Figure 5**). The energy gaps of compounds 1 and **3** are 2.13121 eV and 2.14264 eV, which are the two smaller energy gaps of all the compounds. The compound 2 has the largest energy gap value(2.23788 eV). The HOMO-LUMO transition means that the electron density of compound 2 is transferred from OH to N_3_, and compounds 1, 3, 4, 5 and 6 all have electron density transfer from N_3_ to py. It is only worth noting that the electron density transfer caused by the HOMO-LUMO transition of compounds **5** and 6 is not obvious, and their energy gap is second only to compound 2(2.16985 and 2.18618 eV).

**Table 4:** HOMO–LUMO energy gaps and related molecular properties of the compounds 1, 2, 3, 4, 5 and 6.

Molecular properties	1	2	3	4	5	6
Energies (a.u.)	-1090.81013	-1129.99212	-1129.99031	-1129.99151	-1169.08848	-1169.09029
E_HOMO_ (eV)	-5.64586	-5.77212	-5.58436	-5.57484	-5.55470	-5.50600
E_LOMO_ (eV)	-3.51465	-3.53424	-3.44172	-3.40989	-3.38485	-3.31982
Energy gap (eV)	2.13121	2.23788	2.14264	2.16495	2.16985	2.18618
Ionization Potential(IP)	7.76631	8.04239	7.83384	7.82211	7.78902	7.73630
Electron Affinity(EA)	1.82360	1.59132	1.57894	1.53401	1.43707	1.32066

The analysis of the mono-substituted compounds 2, 3 and 4 shows that the energy gap is 2>4>3. Therefore, the reactivity of these analogs is meta-compound>pair-compound>ortho-compound. The analysis of different numbers of substituents shows that the order of the energy gap of the compounds is 1>3>5. Di-3-picoline compound 5 has the highest reactivity, followed by mono-3-picoline compound 3, and non-picoline compound 1 is the smallest. Similarly, the order of reactivity of different numbers of 4-picoline compounds is also **1**>**4**>**6**. Therefore, as the number of substituents in the compounds increases, the energy gap value of the compounds decreases.

DFT is not enough to satisfy Koopmans Theorem due to the difference between functionals, which results in the calculated vertical ionization potential (VIP) value not approximately equal to -E(HOMO) and vertical electron affinity (VEA) not approximately equal to -E(LUMO) (65). The electron affinity and ionization potential data in **Table 4** are calculated using the LSDA functional method and the SDD basis set. The electron affinities corresponding to compounds 1, 2, 3, 4, 5 and 6 are 1.82360, 1.59132, 1.57894, 1.53401, 1.43707 and 1.32066 eV, and their ionization potentials are 7.76631, 8.04239, 7.83384, 7.82211, 7.778902 and 7.73630 eV respectively. For all the compounds, the electron affinity values of compounds 2, 3 and 4 are almost the same, which may be due to the fact that they are isomers. The value of the electron affinity of the compound 1 is the largest, which means that it has the greatest ability to gain an electron compared with other compounds. The electron affinity of compounds 5 and 6 indicates that they have poor electron-acquiring ability compared with other compounds. Therefore, the order of the ability to obtain electrons is unsubstituted compound>monosubstituted compounds>disubstituted compounds. The order of the ionization potential is 2>3>4>5>1>6. So compound 2 is the least easily ionized among all compounds. The compound 2 is easily reduced by obtaining an electron, which may be the reason for its large dark toxicity. It is worth noting that compound 6 has the largest phototoxic index (PI, PI is the ratio of cytotoxicity after irradiation to cytotoxicity in the dark) (29). The small IP and EA of compound 6 show that it neither loses nor gains electrons easily, and shows good stability in all compounds.

In different mono-substituent compounds 2, 3 and 4, the order of IP and EA values is the 2>3>4. Therefore, the ability of mono-substituted compounds to obtain electrons is ortho-compound>meta-compound>para-compound. The IP values of compounds with different numbers of 3-picoline substituents is 3>5>1, and the IP values of compounds with different numbers of 4-picoline substituents is 4>1>6. The EA values of different numbers of picoline substituents are 1>3>5 and 1>4>6, which indicates that the electron-acquiring ability of the compounds decreases after the number of their picoline substituents increases. The ability to be reduced after obtaining electrons is unsubstituted compound>monosubstituted compounds>disubstituted compounds.

### 3.5 Mulliken and Natural Population Analysis

**Figure 6:**
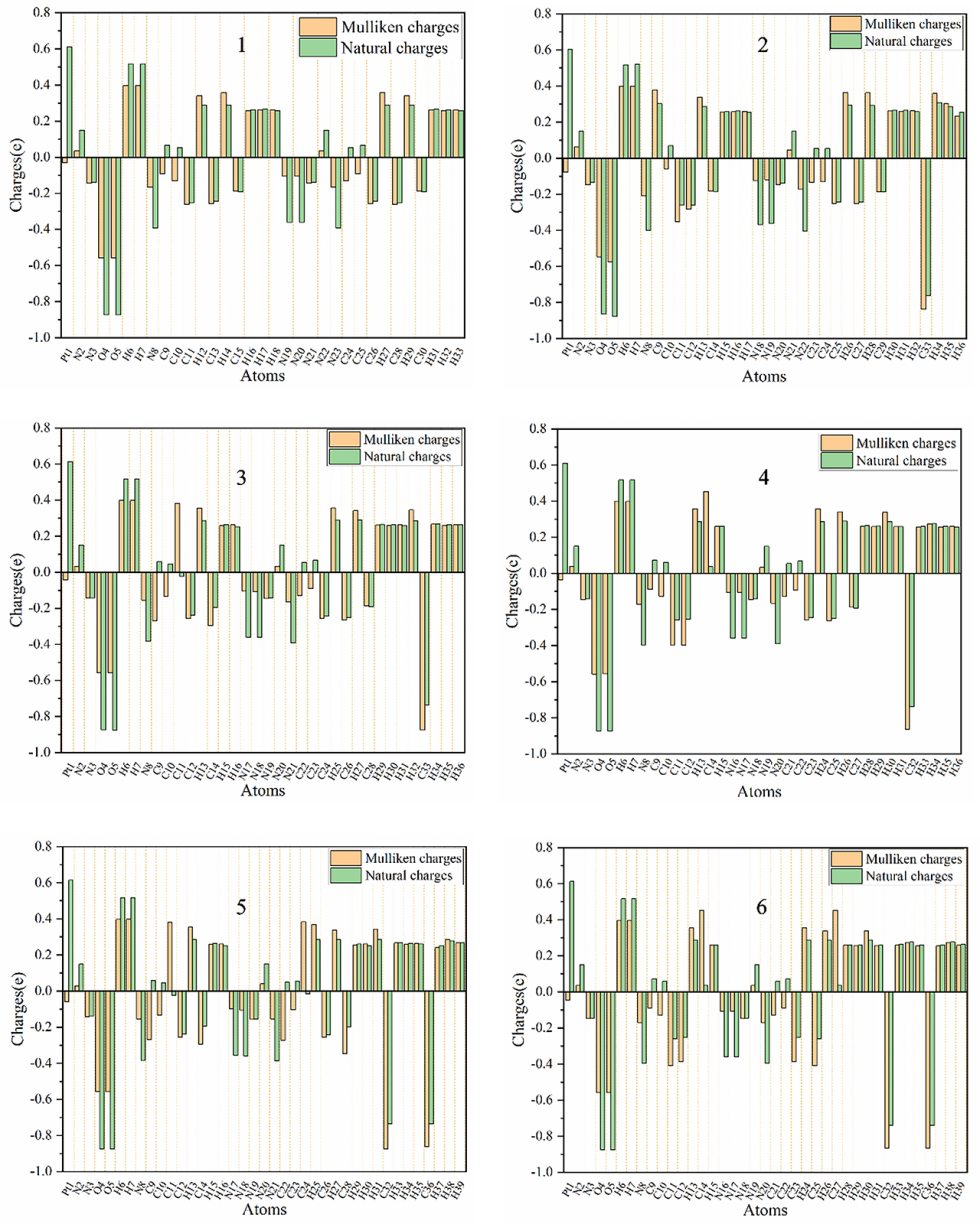
Distribution of calculated Mulliken charges and natural atomic charges for compounds 1, 2, 3, 4, 5 and 6.

Mulliken charges and natural atomic charges analysis is a good way to view the charge distribution between the atoms of a molecule. They can also indicate the positive and negative charges (electron donor and electron acceptor), dipole moment and polarizability of the selected compounds (66, 67). The distributions of Mulliken charges and natural atomic charges calculated using LSDA/SDD levels are shown in **Table 5**. The comparison of the charge distributions of the two methods is shown in **Figure 6**.

**Table 5:** The calculated Mulliken charges and natural atomic charges of compounds 1, 2, 3, 4, 5 and 6.

Atoms	Mulliken charges	Natural charges	Atoms	Mulliken charges	Natural charges	Atoms	Mulliken charges	Natural charges
**1**			**2**			**3**		
Pt1	-0.0300	0.6102	Pt1	-0.0768	0.6045	Pt1	-0.0418	0.6121
N2	0.0354	0.1493	N2	0.0640	0.1500	N2	0.0334	0.1501
N3	-0.1433	-0.1406	N3	-0.1485	-0.1358	N3	-0.1436	-0.1412
O4	-0.5573	-0.8739	O4	-0.5469	-0.8648	O4	-0.5576	-0.8741
O5	-0.5573	-0.8739	O5	-0.5748	-0.8783	O5	-0.5576	-0.8751
H6	0.3980	0.5165	H6	0.3979	0.5182	H6	0.3977	0.5163
H7	0.3980	0.5165	H7	0.3990	0.5200	H7	0.3977	0.5163
N8	-0.1655	-0.3914	N8	-0.2102	-0.3995	N8	-0.1547	-0.3845
C9	-0.0921	0.0665	C9	0.3787	0.3005	C9	-0.2693	0.0579
C10	-0.1301	0.0530	C10	-0.0583	0.0690	C10	-0.1343	0.0461
C11	-0.2619	-0.2508	C11	-0.3535	-0.2592	C11	0.3817	-0.0226
H12	0.3409	0.2885	C12	-0.2832	-0.2606	C12	-0.2565	-0.2385
C13	-0.2564	-0.2444	H13	0.3371	0.2869	H13	0.3555	0.2865
H14	0.3574	0.2881	C14	-0.1842	-0.1845	C14	-0.2960	-0.1952
C15	-0.1866	-0.1911	H15	0.2561	0.2571	H15	0.2586	0.2643
H16	0.2584	0.2629	H16	0.2566	0.2614	H16	0.2620	0.2523
H17	0.2621	0.2652	H17	0.2593	0.2553	N17	-0.1051	-0.3597
H18	0.2623	0.2571	N18	-0.1235	-0.3682	N18	-0.1062	-0.3608
N19	-0.1064	-0.3600	N19	-0.1198	-0.3629	N19	-0.1449	-0.1434
N20	-0.1063	-0.3600	N20	-0.1483	-0.1398	N20	0.0330	0.1493
N21	-0.1434	-0.1406	N21	0.0456	0.1482	N21	-0.1655	-0.3913
N22	0.0356	0.1493	N22	-0.1714	-0.4038	C22	-0.1291	0.0534
N23	-0.1655	-0.3914	C23	-0.1347	0.0530	C23	-0.0913	0.0666
C24	-0.1301	0.0530	C24	-0.1321	0.0526	C24	-0.2572	-0.2450
C25	-0.0921	0.0665	C25	-0.2524	-0.2430	H25	0.3567	0.2880
C26	-0.2564	-0.2444	H26	0.3629	0.2930	C26	-0.2639	-0.2511
H27	0.3574	0.2881	C27	-0.2525	-0.2427	H27	0.3409	0.2885
C28	-0.2618	-0.2508	H28	0.3617	0.2915	C28	-0.1866	-0.1916
H29	0.3409	0.2885	C29	-0.1860	-0.1875	H29	0.2615	0.2648
C30	-0.1866	-0.1910	H30	0.2607	0.2651	H30	0.2581	0.2626
H31	0.2621	0.2652	H31	0.2603	0.2652	H31	0.2617	0.2567
H32	0.2584	0.2629	H32	0.2621	0.2575	H32	0.3445	0.2842
H33	0.2623	0.2571	C33	-0.8387	-0.7641	C33	-0.8735	-0.7354
			H34	0.3576	0.3081	H34	0.2663	0.2676
			H35	0.3024	0.2841	H35	0.2606	0.2644
			H36	0.2337	0.2537	H36	0.2646	0.2619
Atoms	Mulliken charges	Natural charges	Atoms	Mulliken charges	Natural charges	Atoms	Mulliken charges	Natural charges
**4**		**5**				**6**		
Pt1	-0.0370	0.6107	Pt1	-0.0582	0.6140	Pt1	-0.0452	0.6112
N2	0.0373	0.1499	N2	0.0294	0.1501	N2	0.0350	0.1499
N3	-0.1468	-0.1428	N3	-0.1416	-0.1403	N3	-0.1481	-0.1451
O4	-0.5579	-0.8741	O4	-0.5563	-0.8739	O4	-0.5582	-0.8747
O5	-0.5577	-0.8745	O5	-0.5588	-0.8759	O5	-0.5582	-0.8746
H6	0.3976	0.5160	H6	0.3969	0.5155	H6	0.3969	0.5154
H7	0.3974	0.5159	H7	0.3976	0.5161	H7	0.3969	0.5154
N8	-0.1701	-0.3966	N8	-0.1550	-0.3846	N8	-0.1703	-0.3966
C9	-0.0901	0.0721	C9	-0.2687	0.0584	C9	-0.0894	0.0731
C10	-0.1270	0.0594	C10	-0.1325	0.0465	C10	-0.1280	0.0585
C11	-0.3962	-0.2587	C11	0.3813	-0.0231	C11	-0.4084	-0.2598
C12	-0.3973	-0.2528	C12	-0.2574	-0.2392	C12	-0.3862	-0.2526
H13	0.3554	0.2878	H13	0.3542	0.2862	H13	0.3552	0.2876
C14	0.4524	0.0360	C14	-0.2962	-0.1959	C14	0.4524	0.0352
H15	0.2615	0.2605	H15	0.2583	0.2640	H15	0.2605	0.2601
N16	-0.1063	-0.3598	H16	0.2616	0.2520	N16	-0.1063	-0.3595
N17	-0.1065	-0.3601	N17	-0.0968	-0.3549	N17	-0.1063	-0.3595
N18	-0.1442	-0.1426	N18	-0.1055	-0.3585	N18	-0.1481	-0.1451
N19	0.0324	0.1493	N19	-0.1569	-0.1548	N19	0.0350	0.1499
N20	-0.1655	-0.3914	N20	0.0403	0.1511	N20	-0.1704	-0.3966
C21	-0.1296	0.0531	N21	-0.1546	-0.3865	C21	-0.1280	0.0585
C22	-0.0927	0.0664	C22	-0.2722	0.0488	C22	-0.0895	0.0730
C23	-0.2571	-0.2450	C23	-0.1029	0.0549	C23	-0.3867	-0.2527
H24	0.3568	0.2880	C24	0.3830	-0.0155	H24	0.3552	0.2876
C25	-0.2624	-0.2512	H25	0.3694	0.2855	C25	-0.4078	-0.2598
H26	0.3409	0.2885	C26	-0.2557	-0.2443	H26	0.3376	0.2877
C27	-0.1871	-0.1919	H27	0.3380	0.2862	C27	0.4524	0.0352
H28	0.2616	0.2648	C28	-0.3485	-0.1995	H28	0.2606	0.2601
H29	0.2578	0.2624	H29	0.2545	0.2617	H29	0.2576	0.2577
H30	0.3376	0.2877	H30	0.2610	0.2514	H30	0.3376	0.2877
H31	0.2577	0.2582	H31	0.3441	0.2843	H31	0.2576	0.2577
C32	-0.8645	-0.7396	C32	-0.8738	-0.7353	C32	-0.8649	-0.7395
H33	0.2565	0.2606	H33	0.2660	0.2672	H33	0.2585	0.2630
H34	0.2747	0.2758	H34	0.2604	0.2642	H34	0.2743	0.2754
H35	0.2568	0.2615	H35	0.2643	0.2616	H35	0.2542	0.2587
H36	0.2617	0.2566	C36	-0.8631	-0.7362	C36	-0.8649	-0.7395
			H37	0.2410	0.2519	H37	0.2543	0.2588
			H38	0.2869	0.2787	H38	0.2743	0.2754
			H39	0.2663	0.2682	H39	0.2584	0.2628

The calculated natural charge shows that the Pt atoms of the selected compounds have positive charge, and the positive charge is the largest among all the atoms (0.6045-0.6140 e). Among all the compounds, the smallest Pt positive charge, the largest *N*(*Pt-N*_3_) negative charge and the larger R(Pt – N*_N3_*) show that compound 30 is easier to be reduced to Pt(IV) than other compounds (68) All H atoms are also positively charged (0.2514-0.5200 e). The O atoms of the hydroxyl groups of all compounds have negative charge (between -0.8648 e and -0.8783 e). Except for the N(N_3_) atoms in the middle of the azido group have positive charge, all other N atoms(the N(N_3_) atoms at both ends of the azido group, and the N(py) atom of pyridyl group connected to the Pt atom) have negative charge.

The C11, C26 and C28 (corresponding atoms of other compounds) of compound 1 all have negative charge, and its C9, C10, C24 and C25 (corresponding atoms of other compounds) all have positive charges. And the methyl C atoms of compounds 2, 3, 4, 5, and 6 are all negatively charged. And the C_CH3_ atoms of compounds 2, 3, 4, 5 and 6 all have negative charge. The C13 of the compound 1 has positive charge, while the other compounds have negative charge. The charge distribution of the *para*-C atoms of the py group is different between different compounds. The C15 of compound 1 (the corresponding *para*-C atoms of compounds 2, 3 and 5) have negative charge, while the corresponding atoms of compounds 4 and 6 have positive charge. The C30 of compounds 1, 2, 3, 4 and 5 all have negative charge, while the compound 6 has positive charge. Therefore, the main difference between them comes from the addition of a methyl group, which often results in the decrease of the negative charge of the C atom or the conversion of the C atom from negative to positive.

### 3.6 Natural Bond Orbital Analysis

Natural bond orbital(NBO) analysis explains the Lewis structure, bond type, hybridization type, and the strength of the orbital interaction between the electron donor(i) and the electron acceptor(j) (69). In this article, NBO analysis of selected compounds is performed at the LSDA/SDD level. The strength of the i-j interaction is estimated by the magnitude of the second-order perturbation interaction energy E^(2)^, which is derived from the second-order perturbation method:


$${\text{E}}^{\left(2\right)}=\Delta E_{ij}=q_{i}\frac{F{\left(i,j\right)}^{2}}{\epsilon_{i}-\epsilon_{j}}$$

Where *q_i_* is the donor orbital occupancy, *ε_i_* and are the diagonal elements orbital energies, and F*(i,j)* is the off-diagonal NBO Fock matrix element. The larger E^(2)^ indicates the stronger the interaction, the greater the donation tendency between the electron donor and the electron donor, and the greater the degree of conjugation of the entire system. The delocalization of the electron density between the bonded/lone paired NBO orbital and the antibonded NBO orbital corresponds to a stable donor-acceptor interaction (70). The second-order perturbation stability energy E^(2)^ (kcal/mol) of the compound **1** discussed in this article is listed in **Table 6**. The NBO information of other compounds is basically the same as that of the compound 1 (**Table S1**, **Figure S2**). The N(N_3_) bonding donor of compound 11 σ(Pt1-N19) to the anti-bonding acceptor σ*(Pt1-O4), σ*(Pt1-N8) and σ*(Pt1-N19) lead to large σ-σ* stability energy. Their stability energies are 114.62, 105.57 and 43.66 kcal/mol, respectively. The stability energy of the bond donor σ(Pt1-N19) to the anti-bond acceptor π*(N21-N22) is 39.22 kcal/mol. In addition, there is also a strong interaction between the bond donor σ(Pt1-N8) of N(py) and the anti-bond acceptor σ*(Pt1-O4)、σ*(Pt1-N8) and σ*(Pt1-N19). The E^(2)^ are respectively 42.76, 16.27 and 36.74 kcal/mol. Another larger π-π* interaction stability energy related to the degree of conjugation occurs in pyridine. The bond donor π(C26-C30) to the anti-bonding acceptor π*(N23-C24) and π*(C25-C28), their interaction stability energies E^(2)^ are 28.24 and 13.44 kcal/mol. The interaction energy between the bond donor π(N23-C24) and the anti-bond acceptor π*(C25-C28) is 17.11 kcal/mol. In addition, the magnitude of the interaction energy from the lone pair of electrons LP(N19) to π*(N21-N22) is 125.92 kcal/mol, and the large E^(2)^ value shows the electron donation in the N_3_ group.

**Table 6:** Second-order perturbation theory analysis of Fock matrix in NBO basis for compound **1**.

Donor (i)	Types	ED/e	Acceptor(j)		Types	ED/e	^a^ E^(2)^ (kcal/mol)	^b^ E(i)−E(j)(a.u)	^c^ F(i,j)(a.u)
BD(1)Pt1-N8	σ	1.92667	BD*(1)Pt1-O4		σ*	0.42369	42.76	0.93	0.196
BD(1)Pt1-N8	σ	1.92667	BD*(1)Pt1-N8		σ*	0.42866	16.27	0.74	0.108
BD(1)Pt1-N8	σ	1.92667	BD*(1)Pt1-N19		σ*	0.4628	36.74	0.73	0.163
BD(1)Pt1-N19	σ	1.76366	BD*(1)Pt1-O4		σ*	0.42369	114.62	0.75	0.275
BD(1)Pt1-N19	σ	1.76366	BD*(1)Pt1-N8		σ*	0.42866	105.57	0.56	0.229
BD(1)Pt1-N19	σ	1.76366	BD*(1)Pt1-N19		σ*	0.4628	43.66	0.55	0.147
BD(1)Pt1-N19	σ	1.76366	BD*(3)N21-N22		π*	0.34724	39.22	0.28	0.096
BD(2)N23-C24	π	1.77504	BD*(2)C25-C28		π*	0.26323	17.11	0.3	0.065
BD(2)C26-C30	π	1.61949	BD*(2)N23-C24		π*	0.47008	28.24	0.19	0.066
BD(2)C26-C30	π	1.61949	BD*(2)C25-C28		π*	0.26323	13.44	0.23	0.052
LP(2)N19		1.42835	BD*(2)N21-N22		π*	0.55482	125.92	0.13	0.115

a E^(2)^ = means energy of hyper conjugative interaction (stabilization energy).

b E(j) − E(i) - Energy difference between donor and acceptor i and j NBO orbitals.

c F(i,j) is the fock matrix element between i and j NBO orbitals. BD stands for bond, BD* stands for antibonding. The bond corresponding to the σ-bonding orbital is called the σ bond; the bond corresponding to the σ antibonding orbital is called the σ* bond. The bond corresponding to the π-bonding orbital is called the π bond; the bond corresponding to the π antibonding orbital is called the π* bond. σ* represents the σ bond formed by the electron on the antibonding orbital.

## 4 Conclusion

The azido picoline compound is a promising anti-tumor compound. In this work, for the first time, we have shown the structural information and infrared spectrum information of the azido picoline compounds. The predicted structural characteristics can be used as a theoretical basis for screening candidates in the future, and the infrared spectrum information accurately identifies the characteristic absorption peaks of the compounds. HOMO-LUMO, NBO analysis and the distribution of natural atomic charges are calculated to study the electronic properties of these compounds. In the later work, we will explore the interaction between these compounds and small biological molecules.

## Data Availability Statement

The original contributions presented in the study are included in the article/**Supplementary Material**. Further inquiries can be directed to the corresponding author.

## Author Contributions

MM performed the statistical analysis, organized the database and wrote the manuscript. HG contributed to conception and design of the study. All authors contributed to the article and approved the submitted version.

## Funding

This work was financially supported by Natural Science Foundation of Shandong, China [Grant No. ZR2019MC004], the High-end Talent Team Construction Foundation [Grant No. 108-10000318], the Cooperation Project of University and Local Enterprise in Yantai of Shandong Province (2021XDRHXMXK23) and the High-end Full-time Innovative Talent Introduction Foundation "two-hundred plans" of Yantai.

## Conflict of Interest

The authors declare that the research was conducted in the absence of any commercial or financial relationships that could be construed as a potential conflict of interest.

## Publisher’s Note

All claims expressed in this article are solely those of the authors and do not necessarily represent those of their affiliated organizations, or those of the publisher, the editors and the reviewers. Any product that may be evaluated in this article, or claim that may be made by its manufacturer, is not guaranteed or endorsed by the publisher.
