# Supplementary material for: DFT Study on the Substituent Effect of Anticancer Picoline-Diazido-Pt(IV) Compounds
Source: Front Oncol. 2022 Jan 10;11:749178. doi: 10.3389/fonc.2021.749178 (PMC8784384; doi:10.3389/fonc.2021.749178)
Supplement: Supplementary file 1 [file DataSheet_1.docx]

**Supporting Information (SI)**

**DFT Study on the Substituent Effect of Anticancer Picoline-Diazido-Pt(IV) Compounds**

Meilin Mu, Hongwei Gao^*^

*School of Life Science, Ludong University, Yantai, Shandong, 264025, China.*

**Table of contents**

**Figure S1.** The uniform number of the atoms of the compounds **1, 2, 3, 4, 5** and **6**.

**Table S1.** Second order perturbation theory of Fock matrix in NBO basis for compounds **2, 3, 4, 5** and **6**.

**Figure S2.** The interaction energy E^(2)^ between the donor and acceptor of the compounds **1, 2, 3, 4, 5** and **6**.

| **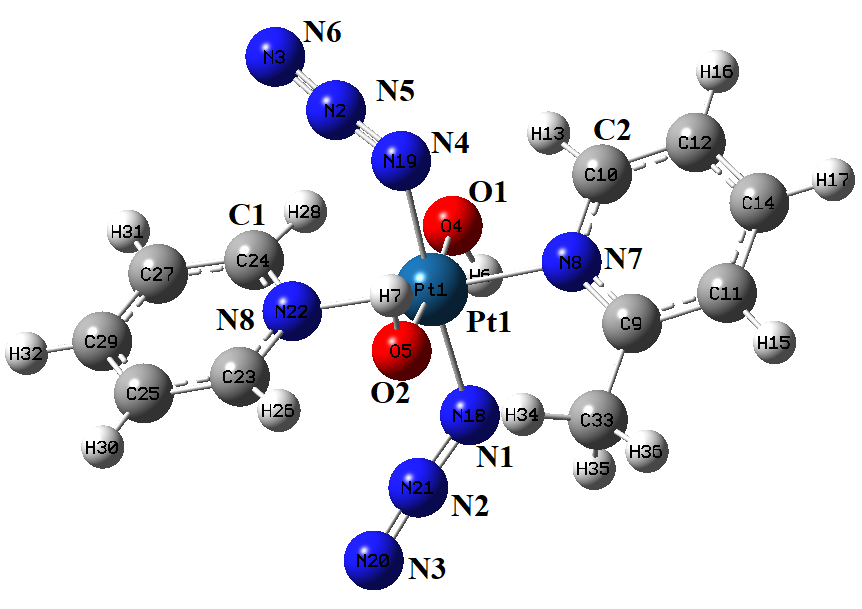Figure S1.** The uniform number of the atoms of the compounds **1, 2, 3, 4, 5** and **6**. |
| --- |

| **Table S1.** Second order perturbation theory of Fock matrix in NBO basis for compounds **2, 3, 4, 5** and **6**. | | | | | | | | | | |
| --- | --- | --- | --- | --- | --- | --- | --- | --- | --- | --- |
| Donor (i) | Types | | ED/e | Acceptor(j) | Types | | ED/e | ^a^ E^(2)^ (kcal/mol) | ^b^ E(i)−E(j)  (a.u) | ^c^ F(i,j)  (a.u) |
| Compound 2 | | | | | | | | | | |
| BD(1)Pt1-N8 | | σ | 1.92486 | BD*(1)Pt1-O4 | | σ* | 0.42782 | 52.33 | 0.94 | 0.218 |
| BD(1)Pt1-N8 | | σ | 1.92486 | BD*(1)Pt1-N8 | | σ* | 0.42759 | 21.93 | 0.72 | 0.122 |
| BD(1)Pt1-N8 | | σ | 1.92486 | BD*(1)Pt1-N18 | | σ* | 0.45588 | 43.64 | 0.69 | 0.173 |
| BD(1)Pt1-N18 | | σ | 1.76429 | BD*(1)Pt1-O4 | | σ* | 0.42782 | 114.27 | 0.8 | 0.283 |
| BD(1)Pt1-N18 | | σ | 1.76429 | BD*(1)Pt1-N8 | | σ* | 0.42759 | 101.26 | 0.58 | 0.227 |
| BD(1)Pt1-N18 | | σ | 1.76429 | BD*(1)Pt1-N18 | | σ* | 0.45588 | 42.57 | 0.55 | 0.145 |
| BD(1)Pt1-N18 | | σ | 1.76429 | BD*(3)N20-N21 | | π* | 0.3466 | 40.11 | 0.28 | 0.097 |
| BD(2)N22-C23 | | π | 1.77678 | BD*(2)C24-C27 | | π* | 0.26266 | 17.42 | 0.3 | 0.066 |
| BD(2)C25-C29 | | π | 1.61583 | BD*(2)N22-C23 | | π* | 0.47585 | 28.79 | 0.18 | 0.067 |
| BD(2)C25-C29 | | π | 1.61583 | BD*(2)C24-C27 | | π* | 0.26266 | 13.57 | 0.23 | 0.052 |
| LP(2)N18 | |  | 1.42813 | BD*(2)N20-N21 | | π* | 0.55631 | 127.93 | 0.13 | 0.116 |
| Compound 3 | |  |  |  | |  |  |  |  |  |
| BD(1)Pt1-N8 | | σ | 1.92587 | BD*(1)Pt1-O4 | | σ* | 0.42292 | 42.74 | 0.93 | 0.195 |
| BD(1)Pt1-N8 | | σ | 1.92587 | BD*(1)Pt1-N8 | | σ* | 0.42815 | 16.2 | 0.74 | 0.107 |
| BD(1)Pt1-N8 | | σ | 1.92587 | BD*(1)Pt1-N17 | | σ* | 0.46298 | 36.7 | 0.72 | 0.162 |
| BD(1)Pt1-N17 | | σ | 1.76338 | BD*(1)Pt1-O4 | | σ* | 0.42292 | 114.11 | 0.75 | 0.275 |
| BD(1)Pt1-N17 | | σ | 1.76338 | BD*(1)Pt1-N8 | | σ* | 0.42815 | 105.03 | 0.56. | 0.228 |
| BD(1)Pt1-N17 | | σ | 1.76338 | BD*(1)Pt1-N17 | | σ* | 0.46298 | 43.23 | 0.55 | 0.146 |
| BD(1)Pt1-N17 | | σ | 1.76338 | BD*(3)N19-N20 | | π* | 0.34804 | 38.99 | 0.28 | 0.096 |
| BD(2)N21-C22 | | π | 1.77444 | BD*(2)C23-C26 | | π* | 0.26332 | 17.15 | 0.3 | 0.065 |
| BD(2)C24-C28 | | π | 1.62026 | BD*(2)N21-C22 | | π* | 0.46909 | 28.15 | 0.19 | 0.066 |
| BD(2)C24-C28 | | π | 1.62026 | BD*(2)C23-C26 | | π* | 0.26332 | 13.42 | 0.23 | 0.052 |
| LP(2)N17 | |  | 1.42728 | BD*(2)N19-N20 | | π* | 0.55628 | 126.33 | 0.13 | 0.115 |
| Compound 4 | |  |  |  | |  |  |  |  |  |
| BD(1)Pt1-N8 | | σ | 1.92654 | BD*(1)Pt1-O4 | | σ* | 0.3474 | 42.54 | 0.93 | 0.195 |
| BD(1)Pt1-N8 | | σ | 1.92654 | BD*(1)Pt1-N8 | | σ* | 0.3474 | 16.16 | 0.74 | 0.107 |
| BD(1)Pt1-N8 | | σ | 1.92654 | BD*(1)Pt1-N16 | | σ* | 0.46225 | 36.63 | 0.73 | 0.162 |
| BD(1)Pt1-N16 | | σ | 1.76373 | BD*(1)Pt1-O4 | | σ* | 0.3474 | 114.38 | 0.75 | 0.275 |
| BD(1)Pt1-N16 | | σ | 1.76373 | BD*(1)Pt1-N8 | | σ* | 0.3474 | 105.24 | 0.56 | 0.229 |
| BD(1)Pt1-N16 | | σ | 1.76373 | BD*(1)Pt1-N16 | | σ* | 0.46225 | 43.7 | 0.55 | 0.147 |
| BD(1)Pt1-N16 | | σ | 1.76373 | BD*(3)N18-N19 | | π* | 0.3474 | 39.1 | 0.28 | 0.096 |
| BD(2)N20-C21 | | π | 1.77434 | BD*(2)C22-C25 | | π* | 0.26367 | 17.14 | 0.3 | 0.065 |
| BD(2)C23-C27 | | π | 1.62033 | BD*(2)N20-C21 | | π* | 0.4693 | 28.14 | 0.19 | 0.066 |
| BD(2)C23-C27 | | π | 1.62033 | BD*(2)C22-C25 | | π* | 0.26367 | 13.43 | 0.23 | 0.052 |
| LP(2)N16 | |  | 1.42768 | BD*(2)N18-N19 | | π* | 0.55622 | 126.38 | 0.13 | 0.115 |
| ^a^ E^(2)^ = means energy of hyper conjugative interaction (stabilization energy).  ^b^ *E(j)*− *E(i)* - Energy difference between donor and acceptor *i* and *j* NBO orbitals.  ^c^ *F(i,j)* is the fock matrix element between *i* and *j* NBO orbitals. | | | | | | | | | | |

| **Table S1.** Continued. | | | | | | | | | | |
| --- | --- | --- | --- | --- | --- | --- | --- | --- | --- | --- |
| Donor (i) | Types | | ED/e | Acceptor(j) | Types | | ED/e | ^a^ E^(2)^ (kcal/mol) | ^b^ E(i)−E(j)  (a.u) | ^c^ F(i,j)  (a.u) |
| Compound 5 | | | | | | | | | | |
| BD(1)Pt1-N8 | | σ | 1.92578 | BD*(1)Pt1-O4 | | σ* | 0.42179 | 42.36 | 0.92 | 0.194 |
| BD(1)Pt1-N8 | | σ | 1.92578 | BD*(1)Pt1-N8 | | σ* | 0.43034 | 15.98 | 0.74 | 0.107 |
| BD(1)Pt1-N8 | | σ | 1.92578 | BD*(1)Pt1-N17 | | σ* | 0.46542 | 36.17 | 0.72 | 0.161 |
| BD(1)Pt1-N17 | | σ | 1.75914 | BD*(1)Pt1-O4 | | σ* | 0.42179 | 115.16 | 0.75 | 0.275 |
| BD(1)Pt1-N17 | | σ | 1.75914 | BD*(1)Pt1-N8 | | σ* | 0.43034 | 105.28 | 0.56 | 0.228 |
| BD(1)Pt1-N17 | | σ | 1.75914 | BD*(1)Pt1-N17 | | σ* | 0.46542 | 43.19 | 0.55 | 0.145 |
| BD(1)Pt1-N17 | | σ | 1.75914 | BD*(3)N19-N20 | | π* | 0.35603 | 40.7 | 0.27 | 0.097 |
| BD(2)N21-C22 | | π | 1.78503 | BD*(2)C23-C26 | | π* | 0.27086 | 16.43 | 0.3 | 0.064 |
| BD(2)C24-C28 | | π | 1.60676 | BD*(2)N21-C22 | | π* | 0.46567 | 27.74 | 0.19 | 0.065 |
| BD(2)C24-C28 | | π | 1.60676 | BD*(2)C23-C26 | | π* | 0.27086 | 14.47 | 0.23 | 0.053 |
| LP(2)N17 | |  | 1.42245 | BD*(2)N19-N20 | | π* | 0.55614 | 122.92 | 0.13 | 0.115 |
| Compound 6 | |  |  |  | |  |  |  |  |  |
| BD(1)Pt1-N8 | | σ | 1.92659 | BD*(1)Pt1-O4 | | σ* | 0.42316 | 40.32 | 0.92 | 0.19 |
| BD(1)Pt1-N8 | | σ | 1.92659 | BD*(1)Pt1-N8 | | σ* | 0.42732 | 14.72 | 0.73 | 0.102 |
| BD(1)Pt1-N8 | | σ | 1.92659 | BD*(1)Pt1-N16 | | σ* | 0.46207 | 34.67 | 0.72 | 0.157 |
| BD(1)Pt1-N16 | | σ | 1.76346 | BD*(1)Pt1-O4 | | σ* | 0.42316 | 114.87 | 0.75 | 0.276 |
| BD(1)Pt1-N16 | | σ | 1.76346 | BD*(1)Pt1-N8 | | σ* | 0.42732 | 105.32 | 0.56 | 0.229 |
| BD(1)Pt1-N16 | | σ | 1.76346 | BD*(1)Pt1-N16 | | σ* | 0.46207 | 44.16 | 0.55 | 0.148 |
| BD(1)Pt1-N16 | | σ | 1.76346 | BD*(3)N18-N19 | | π* | 0.34847 | 39.38 | 0.28 | 0.096 |
| BD(2)N20-C21 | | π | 1.77632 | BD*(2)C22-C25 | | π* | 0.26188 | 17.46 | 0.3 | 0.066 |
| BD(2)C23-C27 | | ^d^ N/A | N/A | BD*(2)N20-C21 | | N/A | N/A | N/A | N/A | N/A |
| BD(2)C23-C27 | | N/A | N/A | BD*(2)C22-C25 | | N/A | N/A | N/A | N/A | N/A |
| LP(2)N16 | |  | 1.42684 | BD*(2)N18-N19 | | π* | 0.55685 | 126.73 | 0.13 | 0.115 |
| ^a^ E^(2)^ = means energy of hyper conjugative interaction (stabilization energy).  ^b^ *E(j)*− *E(i)* - Energy difference between donor and acceptor *i* and *j* NBO orbitals.  ^c^ *F(i,j)* is the fock matrix element between *i* and *j* NBO orbitals.  ^d^ N/A = not applicable | | | | | | | | | | |

**
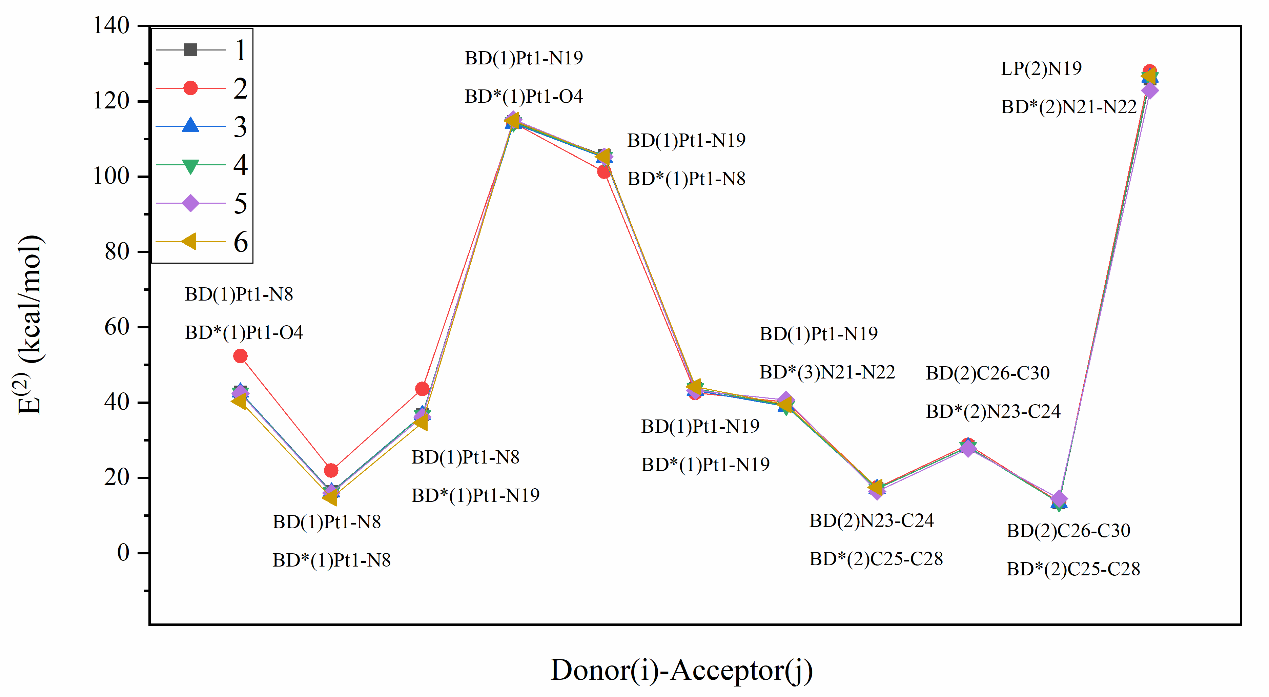
**

**Figure S2.** The interaction energy E^(2)^ between the donor and acceptor of the compounds **1, 2, 3, 4, 5** and **6**.
